# Children’s Health: Sour News for Soy Formula?

**Published:** 2005-05

**Authors:** Julia R. Barrett

Naturally occurring phytoestrogens have been intensively studied for health effects in adults. However, studies of soy formula, which delivers high levels of phytoestrogens to infants, have not extended much beyond ensuring that babies are growing and developing normally. Still, soy formula has been considered a safe alternative to milk-based formulas for some 40 years. Recent studies from the University of Illinois at Urbana–Champaign now show that the soy phytoestrogen genistein can alter intestinal cell proliferation and migration, with unknown effects for infants fed soy formula.

“We are feeding infants soy formula as their sole source of nutrition for the first four to six months of life, a period of time when many systems are immature and undergoing development,” says Sharon Donovan, a professor of nutrition involved in both studies. It is known that infants metabolize genistein and can have some circulating level of the bioactive form.

But whether the effects are good, bad, or even measurable is unknown and fiercely debated. “Why [soy formula] has not received more research attention, I’m not sure,” says Retha Newbold, an NIEHS toxicologist who has investigated the developmental effects of genistein and other estrogenic substances for more than 25 years.

In the first Illinois study, published in the June 2004 *Journal of Nutrition*, researchers exposed human intestinal cells to varying doses of genistein and noted effects on cell numbers, DNA replication, apoptosis, and cell cycle. At low doses, genistein acted as a weak estrogen and stimulated cell growth; at high doses, the compound inhibited proliferation and altered cell cycle dynamics. This biphasic response correlates with how genistein is thought to exert its effects.

In the other study, published in the February 2005 *Pediatric Research*, 24 2-day-old piglets were divided into three dietary groups for eight days, receiving plain sow milk replacer or replacer with either a low or high dose of genistein. The high-dose piglets had circulating concentrations of genistein on par with those of soy formula–fed infants. At 10 days of age, there were no significant differences in weight gain, intestinal length or growth, nutrient uptake, or digestive enzyme activity among the piglets. However, there was a 50% decrease in intestinal cell proliferation and a 20% decrease in cell migration associated with the high genistein dose.

Donovan cautions that it’s premature to draw conclusions about negative or positive effects of infant soy formula. “This is what we see when we look at genistein alone,” she says, “but what happens when you look at a whole soy formula?”

More than 20 million American infants have been fed soy formula in the last 25 years, and their growth and development have been equal to that of infants fed milk-based formula, according to Thomas Badger, director and senior investigator at the Arkansas Children’s Nutrition Center in Little Rock. “If there have been no problems in more than twenty million people exposed to soy formula, then there is no human evidence of a problem,” says Badger.

Still, many researchers believe that more information is needed about the safety of infant soy formula. Phytoestrogen doses comparable to what infants receive through soy formula have been shown to cause cancer in some animal studies if given before puberty. “I think too little is known to conclude that soy formula is safe for the general infant population,” says Newbold.

## Figures and Tables

**Figure:**
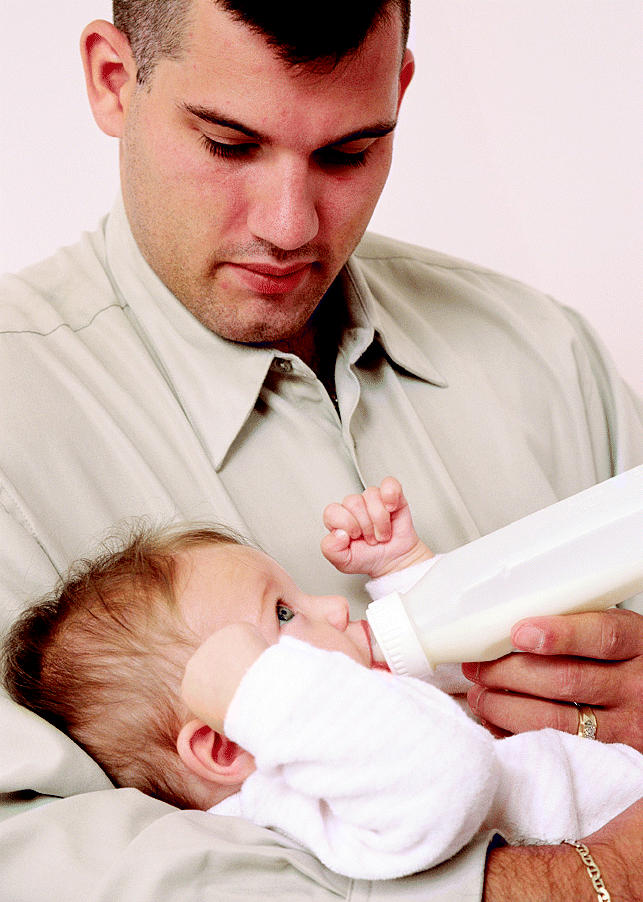
**Formula fitness?** New findings on genistein raise questions about the safety of soy formula.

